# Combustion process of a promising catalyst [Cu(Salen)] for HMX-CMDB propellants†

**DOI:** 10.1039/d3ra04671k

**Published:** 2023-08-30

**Authors:** Wenzhe Ma, Yanjing Yang, Yuxin Jia, Dongxiao Fu, Fengqi Zhao, Kangzhen Xu

**Affiliations:** a Shaanxi Institute of Applied Physical Chemistry Shaanxi 710061 China mawz7344@163.com; b School of Chemical Engineering, Northwest University Xi'an Shaanxi 710069 China xukz@nwu.edu.cn; c Xi'an Modern Chemistry Research Institute Xi'an Shaanxi 710065 China zhaofqi@163.com

## Abstract

Metal organic complexes are regarded as a series of promising combustion catalysts for solid rocket propellants. Their effects on the combustion performance of propellants are closely related to the reaction mechanism. Here, the metal–organic complex Cu(Salen) was investigated as a candidate material for the combustion catalyst of the HMX-added composite modified double-base propellant (HMX-CMDB). The combustion performance of the propellant was found to be evidently enhanced in the presence of Cu(Salen) compared with the propellant samples containing Benzoic-Cu or without catalyst. The addition of Cu(Salen) can improve the burning rate and combustion efficiency of the propellant – and greatly reduce the burning rate pressure index. Analysis shows that the addition of Cu(Salen) can increase the combustion area, flame brightness and combustion surface uniformity of the propellant to a higher degree. The sample can spray more beams of bright filaments on the flat combustion section, and the amount of gas generated by decomposition also greatly increases. In addition, Cu(Salen) shows amazing advantages in improving the surface of the propellant and the temperature gradient of the combustion flame.

## Abstract 2

Metal organic complexes are regarded as a series of promising combustion catalysts for solid rocket propellants.

## Graphical abstract

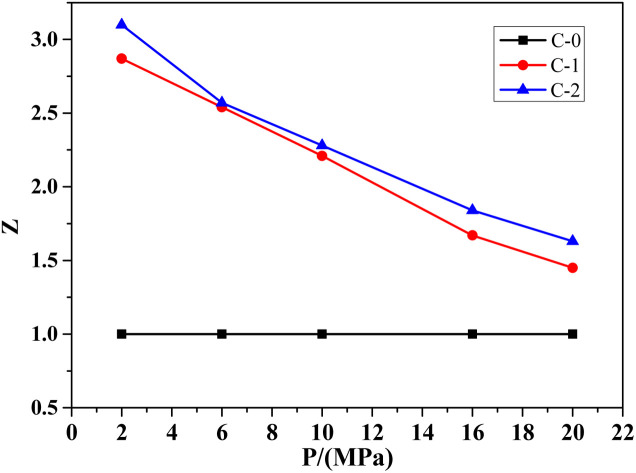

## 1. Introduction

At this stage, national air-to-air missiles, surface-to-air missiles, high-speed kinetic energy bombs and other tactical weapons urgently need to be replaced with new solid propellants with high energy, low characteristic signals, and excellent combustion performances.^1–4^ The addition of strong oxidizing high-energy explosives such as HMX to the double-base propellants provides a way to solve the above problems.^4–7^ At the same time, it also brings new features such as high combustion pressure index, large combustion secondary flame, and seriously unstable combustion.^8–11^ In view of many problems facing the use of propellants, the use of combustion catalysts is one of the effective ways to adjust the combustion performance of solid propellants.^1,12–14^ The combustion catalyst has an impact on the combustion decomposition process of the main components in the propellant formulation through its own special properties, thereby improving its combustion characteristics.^15–17^ For example, carbon nanotubes, with their unique microstructure and excellent chemical activity, can effectively catalyze the thermal behavior of energetic components in propellants. Thus, the burning rate, mechanical characteristics and burning efficiency of CMDB/AP propellants can be successfully enhanced.^18,19^ Since a large amount of primary smoke may be generated during the combustion process of the catalyst, it has a greater impact on the combustion characteristic signal of the propellant.^20,21^ In addition, increasing the amount of catalyst can have an impact on the content of energetic oxides in the propellant composition, resulting in energy loss. Based on this, the efficiency and catalytic performance of the combustion catalyst should be improved as much as possible, so that the addition of the catalyst is beneficial to the combustion of propellant to the greatest extent.^22–24^

Schiff bases, which are commonly considered as privileged ligands for the field of chemical energy, have also attracted great attention in the solid propulsion for both civil and military applications due to their variable structural properties and highly remarkable activity.^25^ Due to their unique properties in the field of catalysis, Schiff bases and their derivatives play an important role in promoting progress of various reactions and changing their reaction selectivity.^26,27^ Because the Schiff base ligand obtained *in situ* has strong structural design flexibility, a variety of Schiff base ligands with different properties due to different structures are derived.^28^ Therefore, the choice of the precursor of the complex is very important for the application performance of the complex in different aspects.^29^ It is believed that *N*,*N*′-bis(salicylidene)ethylenediamine (Salen) has good catalytic, magnetic and biological properties.^30,31^ In particular, its catalytic advantages in olefin hydrogenation, carbene reactions and nitrogen recycling reactions are particularly noticeable.^32^ Generally speaking, the most widely used energetic materials are organic compounds with relatively rich nitrogen content, such as FOX-7 (C_2_H_4_O_4_N_4_), RDX (C_3_H_6_N_6_O_6_) and HMX (C_4_H_8_N_8_O_8_). High-energy materials involve various nitrogen cycle reactions during thermal decomposition and combustion.^33–35^ Based on this, the catalyst with Salen as the precursor is applied to energetic materials, which will have greater potential value for its thermal decomposition and propellant combustion performance.^36^ Schiff bases have a variety of oxidation states that can stabilize the existence of various metals, thereby controlling the performance of the metals in a variety of useful catalytic conversions.^37^ Salen ligand can change the coordinated metal atom without changing its space and electronic environment.^38^ Most coordination metals show strong adaptability and compatibility in the hollow cavity of the Salen ligand.^39,40^ Copper compounds are a kind of combustion catalyst with good ecological safety performance and widely used. Studies have found that copper compounds in the propellant can not only significantly increase the combustion rate, but also have a significant effect on the plateau/meta effect in the medium and high pressure areas. Besides, the copper compound is non-toxic and harmless, and is an ideal green combustion catalyst. Typical copper compounds, such as copper oxide, copper salicylate, copper 2,4-dihydroxybenzoate, copper benzoate and copper adipate, as combustion catalysts, are widely used in the field of double-base propellants.^41,42^

Based on the above point of view, we selected a transition metal copper with higher catalytic activity to chelate with Salen ligand to prepare a new type of combustion catalyst used in solid propellants. It is hoped that this type of catalyst will achieve ideal application effects on the thermal decomposition of energetic materials and the combustion performance of solid propellants.

## 2. Experimental

### 2.1 Materials

All chemicals used for the synthesis were commercially available. Copper nitrate (Cu(NO_3_)_2_, 99%) and NaOH were purchased from Aladdin Inc. Distilled water and ethanol (Xi'an Chemical Reagent Factory, 95%) were used for the preparation of samples. HMX and copper benzoate (Benzoic-Cu) were synthesized in Xi'an Modern Chemistry Research Institute and the purity is over 99%. Nitrocellulose, nitroglycerin and other components in the propellant were also provided by Xi'an Modern Chemistry Research Institute. Salen was synthesized in our institute (Xi'an Modern Chemistry Research Institute) based on the literature and the purity is over 99.5%.^41^

### 2.2 Sample preparation

#### 2.2.1 Cu(Salen) preparation

Salen (5.36 g, 0.02 mol) and NaOH (70 ml, 6 mol L^−1^) were dissolved in ethanol and heated to 55 °C (±2 °C). Then the ethanol solution of Cu(NO_3_)_2_ (20 ml, 0.05 mol L^−1^) was added dropwise. After stirring for 3 h, a dark green precipitate appeared. After filtering, washing and drying, the Cu(Salen) complex was obtained. Yield: 76.5%. Elem anal (%). Calcd for C_16_H_14_N_2_CuO_2_: C, 58.26; H, 4.28; N, 8.49. Found: C, 58.71; H, 4.11; N, 8.84.

#### 2.2.2 Propellant preparation

The traditional double-base propellant manufacturing process is adopted. Through absorbing, water flooding, and light stick calendaring, small medicine strips of *φ* 5 mm × 150 mm were prepared. The compositions of propellant formulations are listed in Table 1.

**Table 1:** The composition of the original formula and the propellant formula with added combustion catalysts

No.	NC/NG (mass%)	HMX (mass%)	Functional components (mass%)	Benzoic-Cu (mass%)	Cu(Salen) (mass%)
C-0	66.3	24	9.7	—	—
C-1	66.3	24	9.7	3	—
C-2	66.3	24	9.7	—	3

### 2.3 Thermal analysis and combustion property evaluation

The catalytic effect of Cu(Salen) composite on the thermal decomposition of HMX and NC/NG were investigated by differential scanning calorimetry (DSC). The experiments were performed using a DSC200 F3 apparatus (NETZSCH, Germany) under a nitrogen atmosphere at a flow rate of 80 ml min^−1^, and the heating rates were 10.0 °C min^−1^ from ambient temperature to 350 °C. The mass ratio of the mixture samples for the DSC tests is 1 : 5 [Cu(Salen) : HMX or NC/NG].

The combustion rate of different propellant samples was tested by the GJB770B-2005 standard “target line method”. Strand burning rates of the propellants were determined at pressures of 2–20 MPa by utilizing the acoustic emission technique. This method involves the combustion of propellant samples with dimensions of 150 mm × 5 mm × 5 mm in a stellar bomb filled with pressurized nitrogen and water. The combustion of propellant samples occurs under water. For evaluating the combustion property, a nickel–chromium alloy wire (*Φ* = 0.15 mm) was utilized for ignition in the experiments. The pressure index *n* is determined by the eqn (1), as followed:

1 *u* = *ap*^*n*^

where *u* is the burning rate, *p* is the pressure, and *a* is a constant.

In order to investigate the combustion flame structure of the CMDB propellants, their flame profiles at different pressures were recorded by a camera using the single frame amplification photography method.

Propellant flameout surface samples are prepared by contact heat conduction flameout method (copper pillar method). The sample with a certain size (2 mm × 5 mm × 15 mm) was pressed on a copper table and filled with nitrogen at different pressures for combustion. The surface morphology of the propellant after the extinguished flame can be observed through the scanning electron microscope (SEM) and recorded with the electronic spectrum.

The combustion wave distributions of propellant samples were obtained using the Π type double tungsten-rhenium thermocouple. The thermocouple with wire size of 25 μm was embedded in the propellant sample (diam = 7 mm, length = 120 mm). In addition, the side of the propellant samples was coated by polyvinyl alcohol for flame retardance. During the combustion process, the burning surface moves gradually to the thermocouple, and finally, the thermocouple gets into the flame zone. Thus, the whole combustion process was recorded and the combustion wave structure from the condensed phase to gas phase was obtained.

## 3. Results and discussion

Copper benzoate (Benzoic-Cu) as a combustion performance modifier is widely used in propellants and has good catalytic effect. In order to evaluate the combustion catalytic performance of Cu(Salen) as a combustion catalyst in HMX-CMDB, Cu(Salen) and Benzoic-Cu were introduced into HMX-CMDB (C-2 and C-3), respectively. In addition, HMX-CMDB sample (C-0) without combustion catalyst was also prepared as comparative samples. The different formulations are listed in Table 1.

The burning rate test was carried out on the prepared propellant samples, and the burning rate pressure index was calculated based on the burning rate data. The data obtained are listed in Table 2.

**Table 2:** Combustion data of HMX-CMDB propellant with different formulations

No.	Catalyst	Burning rate (mm s^−1^)					Pressure exponent	
		2 MPa	6 MPa	10 MPa	16 MPa	20 MPa	10–16	16–20
C-0	—	3.22	7.20	10.37	14.96	17.71	0.72	0.72
C-1	Benzoic-Cu	9.25	18.27	22.85	24.96	25.71	0.19	0.13
C-2	Cu(Salen)	9.99	18.50	23.60	27.52	28.82	0.25	0.21

The combustion data shows that the burning rate of formula C-0 at 2, 6, 10, 16 and 20 MPa is 3.22, 7.20, 10.37, 14.96 and 17.71 mm s^−1^, respectively, and the pressure index is 0.72 at 10 to 20 MPa. The burning rate of the C-1 sample with Benzoic-Cu catalyst at different pressures are 9.25, 18.27, 22.85, 24.96 and 25.71 mm s^−1^, and the pressure index at 10–16 MPa and 16–20 MPa are 0.19 and 0.13, respectively; after adding Cu(Salen) to HMX-CMDB, the burning rate of C-2 sample can reach 9.99, 18.50, 23.60, 27.52 and 28.82, and the pressure index of the sample decrease to 0.25 and 0.21 at 10–16 and 16–20 MPa, respectively. Compared with the propellant sample C-0 without catalyst, the HMX-CMDB sample with combustion catalyst Benzoic-Cu and Cu(Salen) has a greater improvement in combustion performance. Both Benzoic-Cu and Cu(Salen) can increase the combustion rate of the propellant at low pressure (2–10 MPa), and their catalytic effects are basically the same. As the pressure increases, Cu(Salen) has a better performance in improving the combustion performance of HMX-CMDB than Benzoic-Cu. Under 20 MPa, the burning rate of C-2 sample can reach 28.82 mm s^−1^, and its corresponding burning rate pressure index is only 0.25, which provides a reliable guarantee for the further application of HMX-CMDB propellant.

**Fig. 1:**
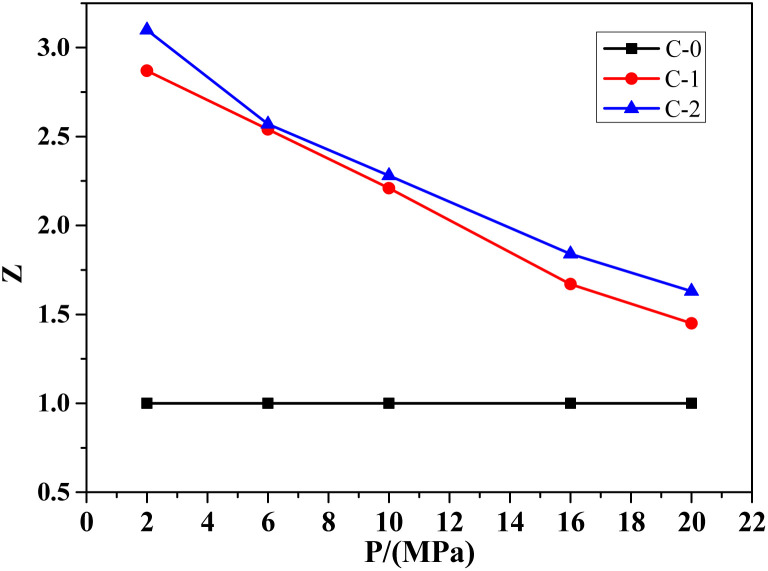
Catalytic efficiency of different combustion catalysts on HMX-CMDB propellant.

Catalytic efficiency is more intuitive in showing the effect of catalyst on combustion rate of the propellant. It can express the catalytic performance of the combustion catalyst during the decomposition process of the propellant, which is the ratio of the combustion rate of samples C-1 or C-2 with the addition of the combustion catalyst to sample C-0 without the catalyst, represented by Z. In addition, based on the formula C-0 without catalyst, the catalytic efficiency curves of Benzoic-Cu and Cu(Salen) for HMX-CMDB are obtained, as shown in Fig. 1.

It can be seen from the Fig. 1 that Benzoic-Cu and Cu(Salen) increase the burning rate of HMX-CMDB propellant at low pressure, which more significantly better than that of high pressure. Especially when Cu(Salen) is used as a combustion catalyst, the catalytic efficiency can reach 3.10, which is very positive for the combustion of solid propellant HMX-CMDB at low pressure. The catalytic efficiency of the catalyst also gradually decreases as the pressure increases. Compared with Cu(Salen), the catalytic effect of Benzoic-Cu is slightly insufficient in terms of the degree of improvement of the burning rate both under low pressure and high pressure. In summary, the addition of Benzoic-Cu and Cu(Salen) can indeed greatly improve the combustion of HMX-CMDB propellant; in addition, compared to the catalytic effect of Benzoic-Cu on HMX-CMDB propellant, Cu(Salen) can improve the burning rate and combustion efficiency of the propellant to a higher degree. Therefore, in the HMX-CMDB propellant, Cu(Salen) is a combustion catalyst with better performance than Benzoic-Cu.

It is well-known that the thermal decomposition properties of energetic compounds are closely related to their combustion properties in propellants. Many researchers have demonstrated that the lower decomposition temperatures of energetic materials would lead to higher burning rates of propellants. The NC/NG composite (NC: Nitrocellulose, NG: nitroglycerine), which contains a large amount of oxidative nitro group (–NO_2_), is the major component of double-base (DB) propellants. Similarly, the nitramine explosive HMX which has a strong oxidizing property plays an indispensable role in improving the propellant energy. Therefore, the thermal decomposition characteristics of HMX and NC/NG are believed to have great influences on the combustion properties of HMX and NC/NG-containing propellants. In order to explore the catalytic mechanism of Cu(Salen) catalyst in the propellant combustion process. Cu(Salen) is physically mixed with the main energetic components in the propellant at a certain mass ratio (Cu(Salen) : NC/NG or HMX = 1 : 5), and the thermal decomposition behavior of the samples are analyzed by DSC.

**Fig. 2:**
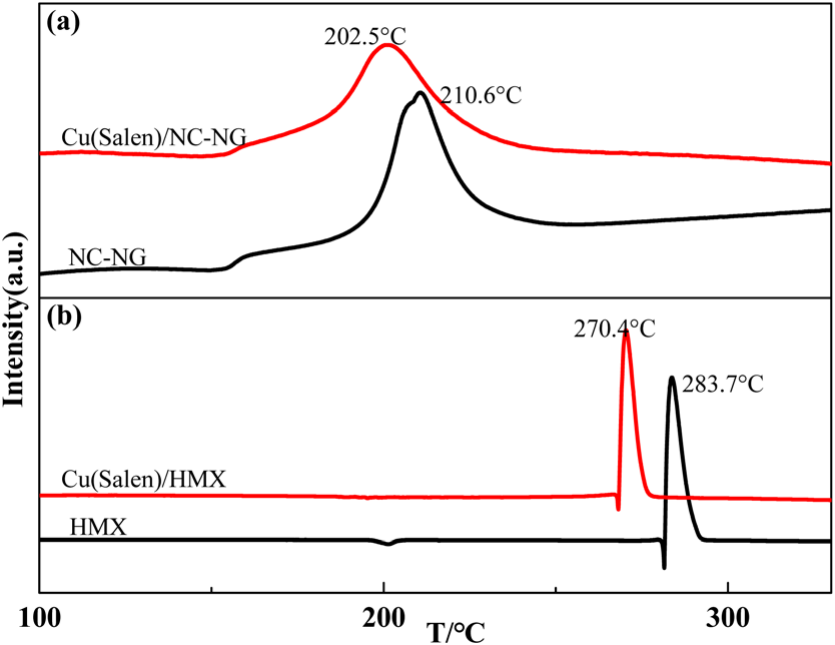
Different promotion effects of Cu(Salen) on NC/NG (a) and HMX (b) thermal decomposition.

The Cu(Salen)–NC/NG or HMX combustion system were subjected to DSC measurements. As shown in Fig. 2(a), only one exothermic event located at 210.6 °C was determined for NC/NG composite, for Cu(Salen)–NC/NG mixture, a similar thermal behavior is found upon heating. The exothermic peak at 202.5 °C should be also attributed to thermal decomposition of NG/NC. As can be seen from Fig. 2(b), the thermal decomposition peak temperature of HMX decreases obviously after adding Cu(Salen). The peak temperature of Cu(Salen)–HMX sample is decreases by 13.3 °C in comparison with that of pristine HMX (283.7 °C at 10 K min^−1^). However, it is noticed that there is a shift to lower temperatures for the exothermic decomposition of NC/NG composite and HMX in the presence of Cu(Salen). According to ref. 38, the addition of metallic Al with high thermal conductivity (230 W m^−1^ K) to NC/NG composite would benefit the heat transfer upon heating and thus reducing its decomposition temperature although no interactions occur between them. Therefore, the introduction of a much higher thermal conductivity of Cu(Salen) (about 80 W m^−1^ K) into NC/NG is the reason behind the decreased decomposition temperature of NC/NG (about 38 W m^−1^ K^−1^). It is also worth noting that, the amounts of energy released from the pristine NC/NG composite and the composite in the mixture were calculated to be 898.3 J g^−1^ and 925.4 J g^−1^, respectively. The results suggest that Cu(Salen) has little effects on the decomposition thermodynamics of NC/NG composite. Based on the above analysis, the reason why the addition of Cu(Salen) can greatly reduce the decomposition temperature of HMX is also because of its the higher thermal conductivity.

Flame structure is a more intuitive method to study the combustion mechanism of propellants, mainly due to the fact that it can obtain important information such as flame height, brightness, thickness of dark region, and shape of combustion surface. The physical structure of HMX-CMDB propellant is uniform and it is mainly made of nitrocellulose, nitroglycerin and HMX fully plasticized. Its combustion flame is a premixed flame, the propagation direction of the combustion wave is one-dimensional, and the chemical reaction kinetics plays a key role in controlling the burning rate.

**Fig. 3:**
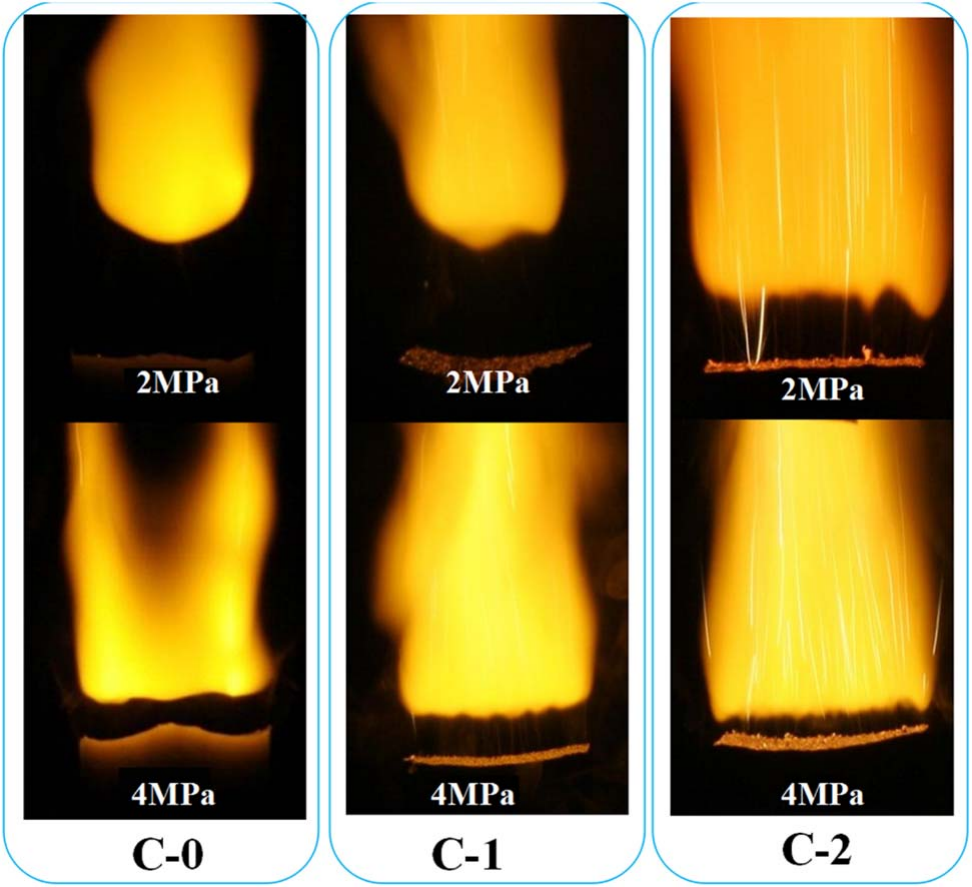
Combustion flame structure of propellant formulations C-0, C-1 and C-2 at different conditions.

The combustion single flames of three solid propellant formulations (C-0, C-1, C-2) at the pressure of 2 MPa and 4 MPa were also recorded and exhibited in Fig. 3.

It can be seen from Fig. 3 that the flame morphology of the catalyst-free HMX-CMDB propellant sample C-0 is relatively loose with thicker bottom dark zone and smaller flame profile, also with few bright spots on the combustion surface. Based on this, it can be inferred that the sample burns insufficiently, which is caused by the incomplete oxidation–reduction reaction in the gas phase zone. By comparison, the flame area of the sample C-1 increases, the brightness increases and it is more concentrated, which is conducive to a more complete combustion of propellant. However, the still very thick dark zone and the combustion section zigzag at 4 MPa, which are not conducive to the stable combustion of the propellant. But for C-2 sample with Cu(Salen) added, the burning flame has a more obvious hissing area, and the bottom dark zone and the upper flame zone are also more obvious. The flame of C-2 sample is brighter and more concentrated than the flames of C-0 and C-1 under the corresponding pressure. At the same time, as the pressure increases, the surface burns more intensely, the thickness of the dark zone is significantly thinner and the brightness of the flame increases. The increase of the bright flame beam from the combustion surface is closer to the combustion surface, and the heat of combustion also increases with the full combustion of the gas phase area, which accelerates the decomposition reaction of the condensed phase, thereby increasing the burning rate.

For both propellants, as expected, the combustion at 4 MPa is more violent than that of 2 MPa, indicating that the high pressure facilitates the combustion of HMX-CMDB propellants. As shown in Fig. 3, the typical “dark zones” of the double base propellant combustion are presented on the photos obtained at both 2 and 4 MPa the thickness of dark zone decreases with the increase of pressure due to the enhanced combustion reactions. It is well-known that, if the temperature of above the propellant is not high enough to trigger the reactions which create light, the “dark zone” would be detected. In the combustion of a typical HMX-CMDB propellant, the energy generated from combustion reaction in the luminous zone is transferred to the dark zone, and then to the propellant samples, which is called “heat feedback”. As for propellant with Cu(Salen), a complete different combustion behaviour is observed. Compared with C-0 and C-1 samples, the dark area of the C-2 sample is obviously thinner under the corresponding pressure. The decrease in the dark zone thickness indicates the increase of heat feedback provided by the gas phase zone to the combustion surface, so the burning rate increases while the pressure increases. Therefore, the combustion of Cu(Salen) with energy output on the burning surface would facilitate “heat feedback” to the propellant, which is believed to be favourable for improving the combustion efficiency of propellant. The flame burning surface of C-2 gradually increases, and the bright clusters of bright filaments appearing on the burning surface in the lower part of the dark zone also gradually increase, and the distribution is relatively uniform, and the combustion section is flat. It is believed that the bright spot is the carbon residue generated after the decomposition of organic energetic materials such as NC and NG or the carbon cluster material generated after the decomposition of the added catalyst. The cavity structure on the surface of these carbon materials will contain many gas components generated by decomposition, which will lead to a more complete oxidation–reduction reaction, thereby improving the heat transfer effect. Therefore, the carbon cluster substances produced after the decomposition of catalyst will improve the catalytic effect and promote the combustion more fully. We found that there are such bright filaments in C-1 and C-2 samples with catalyst added, while the chain-like bright spots of the C-2 sample significantly increase, and the bright filaments are dense and bundled to produce jetting phenomenon submerged in the flame.

It can be seen that the combustion performance of HMX-CMDB propellant is closely related to the degree of clustered bright filament injection and the thickness of dark combustion zone in the flame morphology. The combustion catalyst with better catalytic performance has the characteristics of large combustion surface, high brightness and thin dark area at the bottom, accompanied by a large number of uniformly distributed clusters of bright filaments sprayed out.

**Fig. 4:**
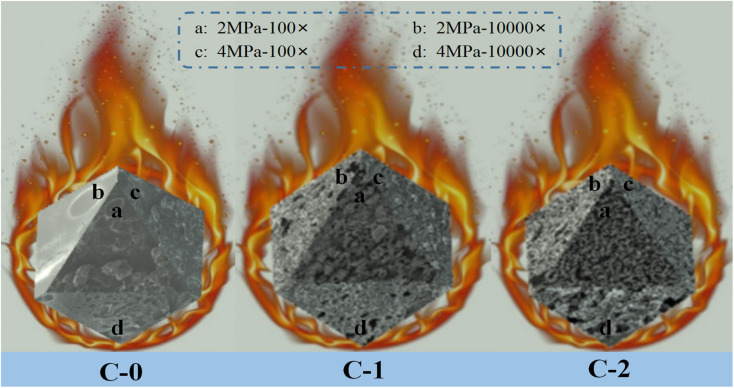
SEM images of flameout surface of propellant formulations C-0, C-1 and C-2 at different conditions.

In order to explore the influence of Benzoic-Cu and Cu(Salen) on the surface morphology of propellant combustion and flameout, the combustion flameout experiment was carried out on C-0, C-1 and C-2. The cross-section of the residue after flameout was observed and recorded using a scanning electron microscope. The SEM of the flameout surfaces of samples C-0, C-1 and C-2 at different magnifications are shown in Fig. 4.

The flameout surface of the blank formulation C-0 shows a porous structure at low power (500×), and the existence of such cavities may be related to the gas stream generated when the propellant burns. It may be that a large amount of gas generated by the thermal decomposition of the propellant's combustion surface area and sub-surface area broke through the molten surface of the system. The movement of the airflow makes the airflow pressure on the surface molten mucosa gradually increase, reaching a limit value at a certain pressure point, and then the air flow breaks through the blockade of the surface molten mucosa; and the air flow gushes out from a certain point of the mucous membrane, thus forming hole-like cavities on the surface of the propellant. However, the flame out surface observed under high magnification (10 000×) has obvious differences under different pressures. The flameout surface at 2 MPa is consistent with the previous analysis results, which is manifested in that as the combustion progresses, a hole-like cavity structure is formed and left on the surface of propellant. As the pressure rises to 4 MPa, the high-magnification SEM shows that the flameout surface of the propellant is a flocculent woven net structure. Although the samples showed the same morphology after burning under different pressure at low magnification, the more microscopic high magnification showed completely different effects. The analysis believes that the reason for this phenomenon is that as the pressure gradually increases, the air flow released by combustion increases sharply, resulting in a greater air pressure on the surface of the propellant mucosa. Therefore, the stress of the molten propellant breaking through the combustion surface are stronger. As the air flow continues to be released, the more release holes are needed after the air flow increases. Gradually, with the larger air flow, the hole-shaped cavity structure is gradually transformed into a flocculent woven net structure, which is more conducive to the release of gas.

For the propellant with Benzoic-Cu catalyst added, a loose and porous carbon framework structure layer can be observed on the surface, and some molten spheres with a particle size greater than 10 μm appear on the carbon framework. The above situation may be due to the decomposition of the Benzoic-Cu catalyst resulting from the high temperature generated by the combustion of the propellant. High temperature can break the organic framework of the complex to generate corresponding metal oxide, which has very high catalytic activity. It is precisely because of the production of such metal oxides that the combustion performance of the solid propellant is improved, and the part of this type of oxide that does not participate in the catalytic decomposition can cause metal melting on the surface of propellant, resulting in the phenomenon shown in the Fig. 4. The extinguishing surface of the formula C-1 with Benzoic-Cu catalyst added can still observe the hole-like cavity structure, which indicates that the thermal decomposition of the HMX/double-base binder system in the combustion surface area and the subsurface area can still occur. The resulting large amount of decomposed gas breaks through the carbon layer remaining on the surface of molten propellant on the combustion surface. As analyzed above, with the increase of pressure, the hole-like structure of the propellant flameout surface becomes more obvious, and the airflow pressure gradually increases, indicating that the combustion decomposition is further accelerated. And more importantly, comparing the flameout surface of C-1 at different pressures, it can be found that there is a significant difference in the degree of looseness of the carbon skeleton components in the propellant surface layer at a pressure of 2 MPa and 4 MPa. Further observation can be found that the propellant surface has better three-dimensionality at 2 MPa, while the flame-out surface material at 4 MPa mostly adheres to the surface of the propellant, which means that as the pressure increases, the gas flow released during combustion gradually increases, causing the carbon skeleton on the surface of the propellant to not be able to adhere well. Therefore, the propellant flameout surface at 4 MPa lacks a part of the carbon material with a three-dimensional framework, which shows a poor three-dimensional effect. The above analysis shows that the addition of the catalyst has a very great significance in promoting the further decomposition of the effective components in propellant during the combustion process.

Compared with the molten spheres that appeared on the carbon skeleton of the flameout surface of C-1, they were not found on the flameout surface of the sample C-2 formulation. The above analysis of this type of molten sphere is considered to be the corresponding metal oxide formed by the decomposition of the metal–organic complex after heating, which is attached to the carbon framework. This type of metal oxide is a highly active material that plays a key catalytic role in solid propellant applications. However, no obvious molten spheres of metal oxides were found on the combustion residue after adding the Cu(Salen) catalyst, which indicates that the amount of catalyst involved in the action increases after Cu(Salen) is added as a combustion catalyst to the HMX-CMDB. The amount of catalyst added in formula C-1 and C-2 is the same (3%), which means that during the combustion process of sample formula C-2, the amount of combustion catalyst involved in the reaction increases, thereby increasing the catalyst efficiency of Cu(Salen) and further improving the combustion efficiency of the solid propellant. A large number of carbon skeletons are distributed on the flameout surface of the blank sample C-0 with small holes and cracks. The carbon skeleton of the combustion residue in the flameout surface morphology of the sample C-2 becomes slender and less after adding Cu(Salen) catalyst. In addition, the distribution of propellant residues on the sample surface is very dense, which means that a large amount of carbon skeleton residues on the surface of the propellant sample has not adhered to the sample surface as the combustion reaction progresses, leaving only a small amount of dense carbon skeleton on the surface. Larger and loose carbon skeleton residues have greater resistance at high airflow pressure, resulting in most of the carbon skeleton residues entering the air with the airflow, leaving only part of the carbon material with smaller skeletons attached to the surface of the flameout residue, forming a very dense network-like material. In addition, the phenomenon exhibited by the propellant flameout surface is more obvious at 4 MPa. The amount of gas decomposed by the propellant due to heating gradually increases with the increase in pressure. The specific manifestation is that the airflow at 4 MPa is several times that at 2 MPa, which results in that the only part of the carbon material with a smaller skeleton attached to the propellant flameout surface is also lost with the released airflow. Based on the above reasons, the three-dimensionality of the flame-out surface of the C-2 sample is weaker at 4 MPa, and it tends to be a two-dimensional layered structure. The sample also found that the size of the surface pores under high pressure is larger than that under low pressure in the electron micrograph with high magnification, which proves that the release of airflow in the propellant has a greater relationship with pressure. And the aperture size of the C-2 sample is larger than that of the samples C-0 and C-1, which further explains that compared with the sample without catalyst and the propellant formulation with Benzoic-Cu catalyst, Cu(Salen) is added as a combustion catalyst to the HMX-CMDB propellant, which can make the propellant have better combustion performance.

**Fig. 5:**
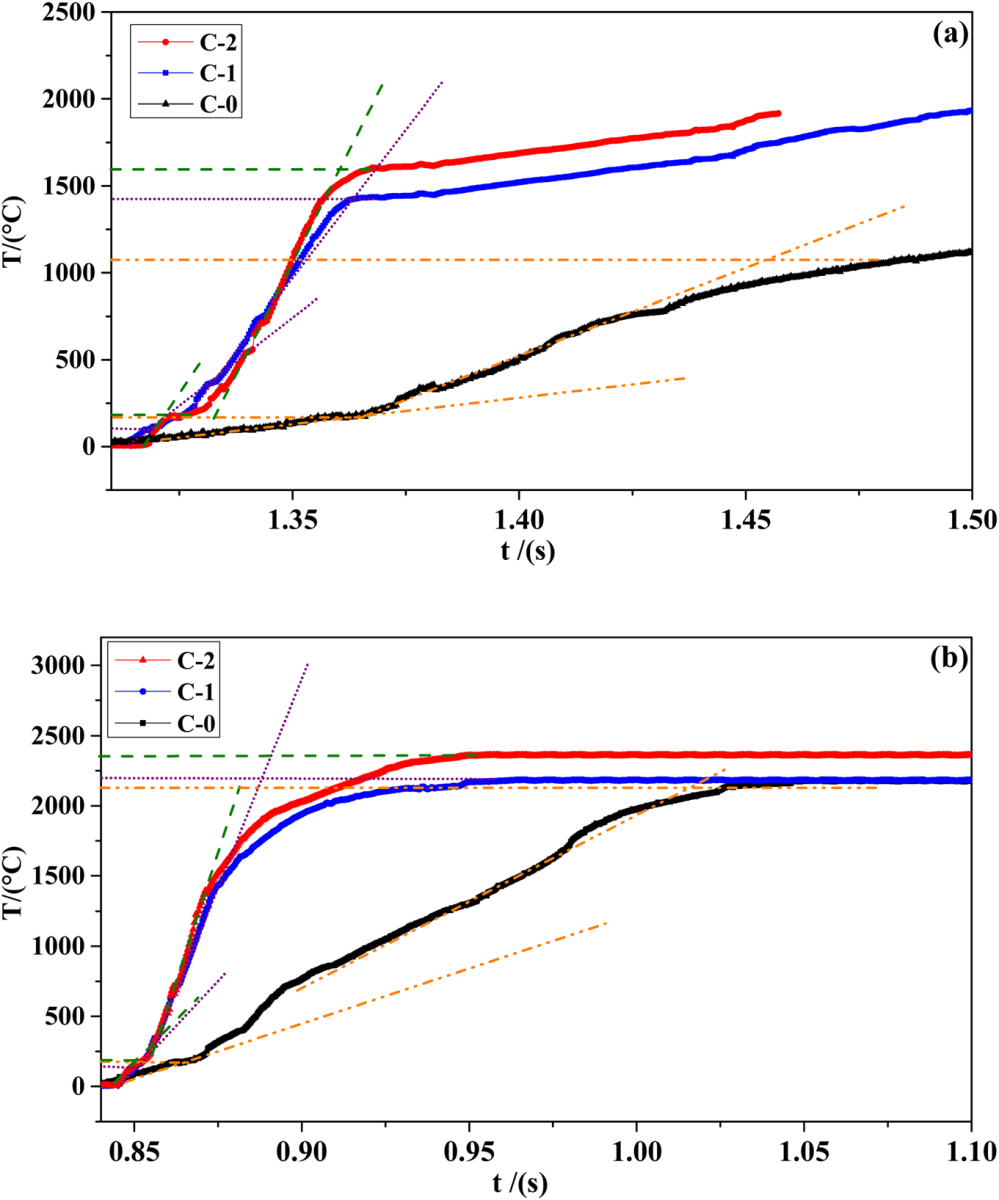
Combustion wave curves of different formulations at 2 MPa (a) and 4 MPa (b).

The combustion wave curve can show the combustion wave structure of the flame during the combustion process indirectly. The combustion wave curves of C-0 formulation without catalyst and the propellant formulations C-1 and C-2 with two different catalysts at different pressures are shown in Fig. 5. The temperature and gradient data corresponding to the combustion wave curve are shown in Table 3.

**Table 3:** Combustion wave temperature gradient table of different HMX-CMDB propellant formulations^a^

No.	*P*/MPa	*T*_s_/°C	*T*_f_/°C	d*T*/d*t* (10^4^ °C s^−1^)↑	d*T*/d*t* (10^4^ °C s^−1^)↓
C-0	2	167	1076	1.11	0.31
	4	172	2139	1.23	0.788
C-1	2	182	1427	3.43	2.02
	4	183	2195	6.22	2.58
C-2	2	203	1603	5.18	3.94
	4	190	2359	7.24	2.49

a *T*_s_/°C in the table represents the surface temperature of the combustion surface; *T*_f_/°C represents the temperature of the combustion flame zone; d*T*/d*t* (10^4^ °C s^−1^)↓ represents the temperature gradient of the condensed phase, d*T*/d*t* (10^4^ °C s^−1^)↑ represents the temperature gradient of the gas phase.

It can be seen from Table 3 that the combustion surface temperature *T*_s_/°C and combustion flame temperature *T*_f_/°C of the three samples of formula C-0, C-1 and C-2 all increase with the increase of pressure. Compared with the blank formula C-0, the surface temperature of C-1 and C-2 are significantly higher. The high temperature promotes the complete decomposition of the energetic organic components in HMX-CMDB, thereby releasing more gas phase products into the gas phase zone, which directly lead to a more violent oxidation–reduction reaction. The above phenomenon confirms that the surface pores of solid propellant become larger with the increase of the pressure in the extinguishing section. In particular, the C-2 formula sample containing Cu(Salen) has a more significant temperature increase compared to the formula C-0, which further illustrates that Cu(Salen) has a more obvious benefits for the deep decomposition of the propellant sample. In addition, it can be seen from the temperature gradient of the gas phase and the condensed phase that the propellant sample with the combustion catalyst has a greater improvement than the blank sample, and the increase in the catalyst Cu(Salen) is more obvious. Generally speaking, the temperature gradient of both the gas phase and the condensed phase increases as the pressure increases. Compared with C-0 and C-1, the sample C-2, which has a better catalytic effect, has a larger change value and range of temperature gradient with the increase of pressure, which also leads to a rapid increase in its burning rate. Based on the above analysis, Cu(Salen) is added to the solid propellant as a combustion catalyst, which leads to a significant improvement for the propellant combustion. Cu(Salen) is expected to replace the widely used copper benzoate as a very effective co-catalyst candidate for solid propellants.

## 4. Conclusions

A copper compound based on the Schiff base (Salen) was studied as a potential combustion catalyst for insensitive solid propellants containing HMX. The addition of Cu(Salen) can improve the burning rate and combustion efficiency of the propellant to a higher degree and greatly reduce the burning rate pressure index compared with the propellant samples containing Benzoic-Cu or without catalyst. Analysis is as follows:

(1) The combustion flame of Cu(Salen) propellant sample is uniform and high in brightness, the combustion surface is large, the dark area is thin, the combustion section is flat, the chain-shaped bright spots are distributed uniformly and a large number of beam-shaped bright filaments are ejected.

(2) Due to the effect of Cu (Salen), the amount of gas generated by the decomposition of the propellant increases, thereby increasing the catalyst efficiency of Cu(Salen).

(3) The increase of the combustion surface temperature *T*_s_/°C and the combustion flame temperature *T*_f_/°C of Cu(Salen)-containing propellant promotes the complete decomposition of the energetic organic components in HMX-CMDB. With the increase of pressure, the temperature gradient of the propellant sample with the catalyst Cu(Salen) has a larger change value and range, which directly leads to a rapid increase in the burning rate.

Based on the above investigation, the Schiff base metal–organic complex has the potential to be a potential combustion catalyst candidate in the future.

## Conflicts of interest

There are no conflicts to declare.

## Supplementary Material

RA-013-D3RA04671K-s001
