# Supplementary material for: Combustion process of a promising catalyst [Cu(Salen)] for HMX-CMDB propellants
Source: RSC Adv. 2023 Aug 30;13(37):25853–61. doi: 10.1039/d3ra04671k (PMC10466475; doi:10.1039/d3ra04671k)
Supplement: RA-013-D3RA04671K-s001 [file RA-013-D3RA04671K-s001.pdf]

Electronic Supplementary Material (ESI) for RSC Advances.
This journal is © The Royal Society of Chemistry 2023
Please do not adjust margins

Combustion mechanism of a promising catalyst [Cu(Salen)] for HMX-CMDB propellant

Received 00th January 20xx,
Accepted 00th January 20xx

DOI: 10.1039/x0xx00000x

Wenzhe Ma[a,b], Yanjing Yang[c], Yuxin Jia [b], Dongxiao Fu [b], Fengqi Zhao*[c],Kangzhen Xu*[b]

Supporting Information

Characterization of Cu(Salen)

Select single crystals of size 0.12×0.08×0.15 mm3 single crystal was subjected to XRD single crystal diffraction testing using single crystal diffractometer. The specific process is as follows: ψ-ω Scanning method, experimental temperature is -173.15 K, Collect data within the scanning range of θ at 2.825-26.393. The single crystal structure data of Cu (Salen) and its refined results are listed in Tables S1.

Table S1 Crystallographic date and structure determination details for Cu(Salen)

Compound	Cu(Salen)
Chemical formula	$C_{16}H_{14}N_2CuO_2$
Formula weight (g mol^{-1})	329.83
Temperature (K)	-173.15
Crystal system	Monoclinic
Space group	*C12/c1(15)*
a (Å)	26.575(3)
b (Å)	6.8984(5)
c (Å)	14.5506(12)
β (°)	97.620(3)
Volume (Å^3)	2643.93(40)
Z	8
Dcalc (g cm^{-3})	1.65712
Absorption coefficient (mm^{-1})	1.657
F(0 0 0)	1352
θ range (°)	2.825 - 26.393
Index ranges	-33≤h≤32, -8≤k≤8, -18≤l≤15
Reflections collected	13357
Reflections unique	2673
Goodness-of-fit on F^2	1.118
Final *R* indices [I > 2σ(I)]	R_1 = 0.0685, wR_2 = 0.1962
R indices (all data)	R_1 = 0.0796, wR_2 = 0.2032
Largest diff. peak and hole (e Å^{-3})	1.834 and -1.000

[a] Shaanxi Institute of Applied Physical Chemistry, shaanxi, 710061, China,E-mail: mawz7344@163.com
[b] School of Chemical Engineering, Northwest University, Xi'an, shaanxi 710069, China.E-mail: xukz@nwu.edu.cn
[c] Xi'an Modern Chemistry Research Institute, Xi'an, shaanxi 710065, China .E-mail: zhaofqi@163.com

The molecular structure and one-dimensional structure of Cu (Salen) are shown in Fig. S1, and its 3D molecular stacking structure is shown in Fig. S2. The partial bond length and bond angle parameters of Cu (Salen) molecule are listed in Tables S2 and S3, respectively.

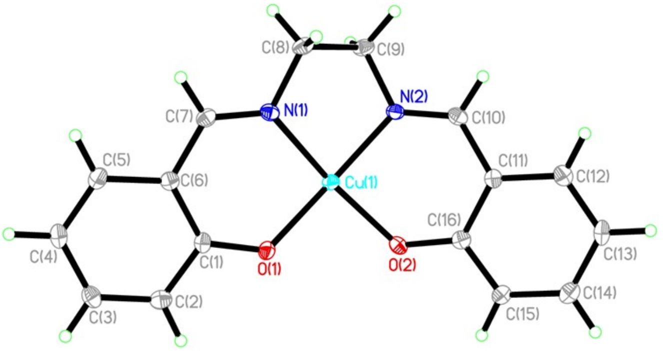

Figure.S1 Crystal structure of Cu(Salen)

The X-ray single crystal diffraction study shows that the compound Cu (Salen) is a Monoclinic crystal system, its spatial structure is C12/c1 (15), and each unit cell contains 24 molecules. As shown in Figure.S1, Cu(Salen) molecules are

Please do not adjust margins

folded and arranged in a wavy manner in space, with each copper atom coordinating with Salen molecules. The copper atom forms a relatively stable planar four coordinated structure with two N atoms [Cu1-N1 (1.713) and Cu1-N2 (1.705)] and two O atoms [Cu1-O1 (1.719) and Cu1-O2 (1.728)] from the Salen ligand.

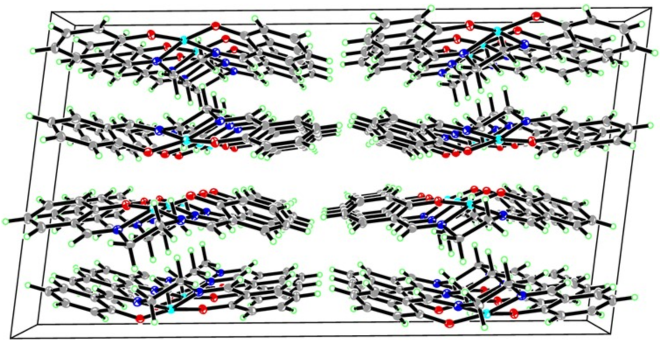

Figure.S2 3D packing diagram structure of Cu(Salen)

From the 3D molecular stacking diagram in Fig.S2, it can be seen that Cu (Salen) is arranged in a layered wave like manner in the three-dimensional spatial system. There is no obvious connection between the molecules in the adjacent columns of the monolayer, and no significant force has been observed between the molecular layers. The interaction between its molecules in the unit cell may be connected by Van der Waals force. The two-dimensional network structure formed by different molecules forms a compact stacking structure through the connection of Van der Waals force, thus forming a three-dimensional and more stable compound.

Table S2 Selected bond lengths(Å) of Cu(Salen)

Bond	Lengths/Å	Bond	Lengths/Å
Cu(1)-O(1)	1.919(5)	C(16)-C(11)	1.431(10)
Cu(1)-O(2)	1.939(5)	C(1)-C(6)	1.424(10)
Cu(1)-N(2)	1.955(6)	C(1)-C(2)	1.425(11)
Cu(1)-N(1)	1.959(6)	C(11)-C(12)	1.397(10)
Cu(1)-O(2)	2.362(5)	C(2)-C(3)	1.370(11)
O(1)-C(1)	1.306(8)	C(15)-C(14)	1.372(11)
O(2)-C(16)	1.316(9)	C(6)-C(5)	1.408(10)
O(2)-Cu(1)	2.362(5)	C(6)-C(7)	1.450(10)
N(2)-C(10)	1.278(10)	C(9)-C(8)	1.507(10)
N(2)-C(9)	1.473(9)	C(12)-C(13)	1.384(11)
N(1)-C(7)	1.282(10)	C(14)-C(13)	1.400(12)
N(1)-C(8)	1.484(9)	C(3)-C(4)	1.394(12)
C(10)-C(11)	1.455(10)	C(5)-C(4)	1.377(11)
C(16)-C(15)	1.396(10)		

Table S3 Selected bond angles (°) of Cu(Salen)

Please do not adjust margins

Please do not adjust margins

Bond	Angles/(°)	Bond	Angles/(°)
O(1)-Cu(1)-O(2)	91.5(2)	O(2)-C(16)-C(11)	123.7(7)
O(1)-Cu(1)-N(2)	170.6(2)	C(15)-C(16)-C(11)	116.6(7)
O(2)-Cu(1)-N(2)	90.9(2)	O(1)-C(1)-C(6)	125.4(7)
O(1)-Cu(1)-N(1)	92.6(2)	O(1)-C(1)-C(2)	118.2(7)
O(2)-Cu(1)-N(1)	169.7(2)	C(6)-C(1)-C(2)	116.4(6)
N(2)-Cu(1)-N(1)	83.6(3)	C(12)-C(11)-C(16)	120.5(7)
O(1)-Cu(1)-O(2)	93.55(19)	C(12)-C(11)-C(10)	116.8(7)
O(2)-Cu(1)-O(2)	85.3(2)	C(16)-C(11)-C(10)	122.7(7)
N(2)-Cu(1)-O(2)	95.7(2)	C(3)-C(2)-C(1)	121.9(7)
N(1)-Cu(1)-O(2)	103.9(2)	C(14)-C(15)-C(16)	122.8(7)
C(1)-O(1)-Cu(1)	126.5(5)	C(5)-C(6)-C(1)	120.3(7)
C(16)-O(2)-Cu(1)	126.4(5)	C(5)-C(6)-C(7)	117.0(7)
C(16)-O(2)-Cu(1)	110.2(4)	C(1)-C(6)-C(7)	122.7(7)
Cu(1)-O(2)-Cu(1)	94.7(2)	N(2)-C(9)-C(8)	106.8(6)
C(10)-N(2)-C(9)	120.9(6)	N(1)-C(8)-C(9)	108.6(6)
C(10)-N(2)-Cu(1)	127.2(5)	N(1)-C(7)-C(6)	124.3(7)
C(9)-N(2)-Cu(1)	111.5(5)	C(13)-C(12)-C(11)	120.8(7)
C(7)-N(1)-C(8)	119.8(6)	C(15)-C(14)-C(13)	120.2(7)
C(7)-N(1)-Cu(1)	127.2(5)	C(2)-C(3)-C(4)	121.0(7)
C(8)-N(1)-Cu(1)	112.9(5)	C(4)-C(5)-C(6)	121.3(7)
N(2)-C(10)-C(11)	124.2(7)	C(5)-C(4)-C(3)	119.0(7)
O(2)-C(16)-C(15)	119.8(7)	C(12)-C(13)-C(14)	119.2(7)

This journal is © The Royal Society of Chemistry 20xx

Please do not adjust margins

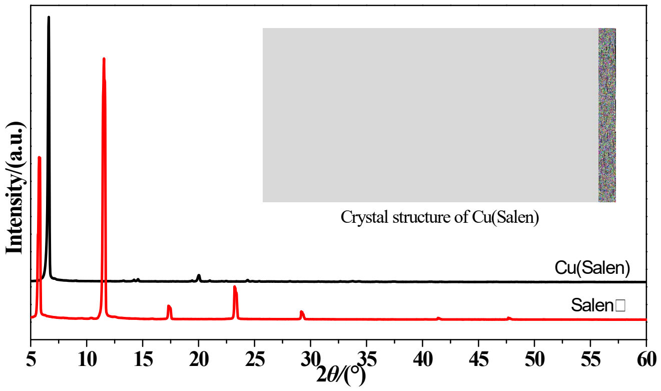

Figure.S3 XRD patterns of Cu(Salen) and Salen ligand

The XRD spectra of the prepared Cu (Salen) complex were obtained and compared with the pure Salen ligand. As shown in Figure.S3, no diffraction characteristic peaks belonging to the Salen ligand were observed on the XRD spectrum of Cu (Salen), indicating that there were no Salen residues in the prepared Cu (Salen) complex samples. Salen has fully participated in the reaction and generated a Cu (Salen) complex. In the XRD spectrum, the new characteristic peaks appearing at 6.65, 14.57, 20.01, 24.36, 28.09, 33.68, and 34.31℃ are attributed to Cu(Salen), which is consistent with literature reports and confirms that the compound has been successfully synthesized with high purity. In addition, the high signal-to-noise ratio of XRD images indicates that Cu (Salen) has good crystallinity.

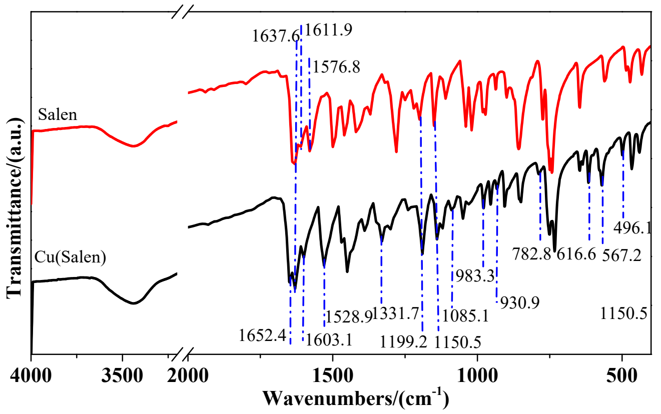

Figure.S4 FT-IR spectrum of the Salen ligand and Cu(Salen)

The structural characteristics of Cu (Salen) were further studied using FT-IR. As shown in Figure.S4, two tensile vibration Absorption band of bridged carbon nitrogen double bond (1150.5 cm^{-1}) and carbon nitrogen single bond (1637.6 cm^{-1}) are detected in the characteristic spectra of Salen ligand and Cu (Salen), which indicates that bridged ethyl in Salen ligand is not involved in coordination. On the other hand, the two phenolic hydroxyl absorption peaks at 1576.8 and 1611.9 cm^{-1} in Salen merge into one (1603.1 cm^{-1}) absorption peak in Cu (Salen), which is caused by the coordination between-OH in Salen ligand and Cu ions. After the formation of the complex, the characteristic bands at 1652.4, 1528.9, 1331.7, 1085.1, 983.3, and 930.9 cm^{-1} are believed to originate from the Cu-N bond in Cu (Salen). In addition, the absorption peaks at 782.8, 616.6, 567.2 and 496.1 cm^{-1} are attributed to the Cu-O Coordinate covalent bond, which indicates that the transition metal in the complex is coordinated with the phenolic hydroxyl group.

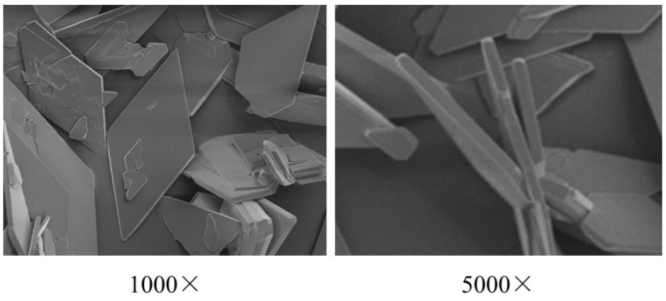

Figure.S5 SEM images of Cu(Salen)

The morphology and microstructure of Cu (Salen) were characterized using SEM. Unlike the Salen sample, which is composed of irregular particles with a diameter of 1-3 um and exhibits agglomeration, the microstructure of Cu (Salen) exhibits a regular and rhombic sheet like structure. The length of the sheet observed on the image is tens of micrometers, while the thickness is several micrometers. Moreover, the layered structure surface of Cu (Salen) sample is relatively smooth. As mentioned above, Cu (Salen) has a two-dimensional layered structure, and these layers are Tight junction with each other through intermolecular forces. The layered crystal structure of Cu (Salen) is believed to be closely related to its morphology.
